# Concise, Simple, and Not Wrong: In Search of a Short-Hand Interpretation of Statistical Significance

**DOI:** 10.3389/fpsyg.2018.02185

**Published:** 2018-11-13

**Authors:** Jeffrey R. Spence, David J. Stanley

**Affiliations:** Department of Psychology, University of Guelph, Guelph, ON, Canada

**Keywords:** significance tests, science communication, science practice, *p*-value, evidence – based practice

## Abstract

One challenge when communicating science to practitioners and the general public is accurately representing statistical results. In particular, describing the meaning of statistical significance to a non-scientific audience is especially difficult given the technical nature of a correct definition. Correct interpretations of statistical significance can be unintuitive, nuanced, and use unfamiliar technical language. As a result, when researchers are tasked with providing short and understandable interpretations of statistical significance it can be tempting to default to convenient but incorrect interpretations. In the current paper, we offer a concise, simple, and correct interpretation of statistical significance that is suitable for communications targeting a general audience.

## Introduction

For researchers in applied fields like industrial/organizational (I/O) psychology that follow the scientist-practitioner model, it is important to be able to disseminate knowledge and communicate science to non-scientific audiences. One challenge often faced by researchers is effectively communicating what statistical significance means. Imagine that you submit an article about your latest study to a popular press publication and the editor returns some edits. One sentence has been changed from, “All of the results were statistically significant” to, “All of the results were statistically significant (indicating that the results were not likely due to chance).”

Do you approve, reject, or modify the edit? Approving it means you sign off on adding an incorrect interpretation of statistical significance. Rejecting it means that you leave it up to readers to know or figure out for themselves what statistical significance means. Modifying it means that you have the difficult task of providing an easy-to-read, but correct, definition of statistical significance for a general audience. When faced with this trilemma, it may be easy to default to a correct sounding albeit incorrect interpretation of statistical significance. Our goal is to help researchers who need to communicate science to non-scientific audiences by providing a concise and easy to understand interpretation of statistical significance that is correct.

In order to effectively disseminate research findings to a general audience, researchers are tasked with simplifying and succinctly describing their results and conclusions. Given the ubiquity of statistical significance, the dissemination process may involve explaining what statistical significance means to a general audience – including managers, executives, lawyers, and journalists. Providing an intelligible and concise explanation of statistical significance can be hard to do without falling prey to common fallacies and misinterpretations (see Kline, 2009 for a review). Providing incorrect interpretations of statistical significance is misleading and perpetuates misunderstandings. Failing to provide an explanation is uninformative and can cause readers to insert their own idiosyncratic misinterpretations. Providing a technically accurate but unintelligible definition reduces the effectiveness of the communication and is ultimately counterproductive to the goal of disseminating scientific results.

Accurately interpreting statistical significance is not easy – history and research show that significance testing is notorious for being misunderstood (e.g., Nickerson, 2000). Correct definitions of statistical significance tend to use technical vocabulary that is also quite nuanced, such that an omission, word inversion, or typo may change a correct definition into a violently incorrect one (see Kline, 2004 for a review). Spotting counterfeit definitions can be so difficult that even those with formal training on the subject of statistical significance can have difficulty distinguishing correct from incorrect definitions and often make interpretational errors (e.g., Haller and Krauss, 2002; Lecoutre et al., 2003; Hoekstra et al., 2006; Castro Sotos et al., 2009).

One implication of these issues is that if a researcher is tasked with providing an understandable definition of statistical significance it can be easy to default to inaccurate definitions and commonly used fallacies. Notably, commonly used fallacies and misinterpretations (Kline, 2004, 2009) have something in common (other than being incorrect): they are often shorter and simpler than correct definitions. Examples of such misinterpretations include: “there is a low probability that the result was due to chance,” “there is less than a 5% chance that the null hypothesis is true,” or “there is a 95% chance of finding the same result in a replication.” What if there was an equally short, simple, and understandable interpretation of statistical significance that was correct?

## Some Background

Since its introduction nearly 90 years ago, null hypothesis significance testing (NHST) has been the most widely used method for statistical analysis in psychology (Nickerson, 2000). Its popularity and longevity may only be rivaled by the magnitude of persistent criticism it has received since its introduction (e.g., Fisher, 1925; Pearce, 1992). Criticisms of NHST have been numerous and have targeted various aspects of the method and its application with little reprieve since it was introduced (see Berkson, 1938; Rozeboom, 1960; Bakan, 1966; Carver, 1978; Nickerson, 2000; Wagenmakers, 2007). NHST has been criticized for its assumptions (e.g., the appropriateness of assuming null hypothesis is true; Clark, 1963; Bakan, 1966; Lykken, 1968; Nickerson, 2000), its reasoning and faulty logic (e.g., how it is the marriage of two incompatible procedures, Berkson, 1942; Berger, 2003; Hubbard and Bayarri, 2003), its standards (e.g., arbitrary nature of *p* < 0.05) its underlying statistical orientation (e.g., frequentist versus Bayesian; Bayarri and Berger, 2004; Efron, 2005; Wagenmakers, 2007), its inaccurate and misleading nomenclature (e.g., it has nothing to do with testing hypotheses; Nunnally, 1960; Bolles, 1962), its utility (e.g., significance testing does not provide useful information; Lykken, 1968). Criticisms of the procedure have occasionally culminated in recommendations to ban NHST (Carver, 1993; Schmidt, 1996; Hunter, 1997). A review of the history of NHST criticisms reveals that researchers’ misunderstanding, misinterpretation, and misapplication of the technique is not only common but is also a contributing factor leading to other criticisms (e.g., Bakan, 1966; Carver, 1978; Cohen, 1994).

## A Brief History of Misinterpretations

For as long as it has been used, NHST has been criticized for being defined or interpreted incorrectly. Bakan (1966) stated “The psychological literature is filled with misinterpretations of the nature of the test of significance” (p. 428). At the time he even caveated his article noting that “What will be said in this paper is hardly original” (p. 423). Giving credence to Bakan’s observation that he was not saying anything new, Rozeboom (1960) critiqued the application and misinterpretation of NHST by psychologists noting that NHST had “attained the status of a religious conviction” (p. 416). In the same year, Nunnally (1960) referred to NHST as “misused and misconceived” (p. 642).

After a decade or so passed since Bakan’s paper, Carver (1978) noted that not much had changed with respect to the application and interpretation of NHST. He then outlined what he referred to as “fantasies” about statistical significance. He identified three fantasies, odds-against chance fantasy, replicability fantasy, and valid research hypothesis fantasy, which categorized incorrect inferences that were drawn from significance tests.

As the years passed, misinterpretations of significance testing continued and Cohen (1994) revisited problems with NHST, in his paper *The Earth is Round (p < 0.05)*. At the outset of the paper, he explicitly notes that the ideas he was expressing were not original but were said many times before. He then identifies that the problem with NHST lies in its misuse and misinterpretation by researchers. By the late 1990s, the American Psychological Association (APA) formed a *Task Force on Statistical Inference* in 1996 (Wilkinson and Task Force on Statistical Inference, 1999) to “elucidate some of the controversial issues surrounding the applications of statistics including significance testing and its alternatives…”^1^. Ultimately, changes were made to the APA publication manual that were recently re-affirmed in the APA’s journal article reporting standards (Appelbaum et al., 2018). Unfortunately, the issues surrounding the use and interpretation of NHST have persisted.

Nickerson (2000), comprehensively reviewed NHST in paper subtitled “A review of an old and continuing controversy.” A major component of the paper consisted of outlining a series of misconceptions about NHST. Recommendations were made, but, once again, little appeared to change. Additional criticisms were published (e.g., Kline, 2004; Schwab et al., 2011; O’Connor, 2017), then the replication crisis struck psychology (Pashler and Harris, 2012; Pashler and Wagenmakers, 2012). Because of the replication crisis, misinterpretations and misapplications of NHST were once again under fire by methodologists (e.g., Cumming, 2014) and, in 2016, the American Statistical Association issued a statement on NHST, to directly address the misuse and misunderstanding of NHST. The American Statistical Association’s statement noted, “While the *p*-value can be a useful statistical measure, it is commonly misused and misinterpreted (Wasserstein and Lazar, 2016, p. 131).” Indeed, many researchers tend to incorrectly believe a *p*-value indicates effect size as well as many of the interpretational fallacies such as *inverse probability* and the *odds-against-chance* portrayals (Kline, 2009; Badenes-Ribera et al., 2015). Moreover, researchers vastly underestimate the extent to which *p*-values vary from study to study (Lai et al., 2012).

## A Correct Interpretation of Statistical Significance

But what does it mean for something to be statistically significant? Many researchers who have been formally educated on the subject, and some textbooks will (incorrectly) tell you that statistical significance means that the odds that a result happened due to chance is small – specifically, in most cases, that the odds are less than five percent (Dimova et al., 2017). This is a nice, simple, easy to understand interpretation that aligns with a common-sense interpretation of the words “statistical” and “significant” placed side-by-side. Unfortunately, interpreting statistical significance in this way is incorrect and corresponds to the “odds against chance” fallacy (Kline, 2009).

Statistical significance refers to the conditional probability of hypothetical data. In the vast majority of cases where significance testing is used, a researcher starts with the assumption that there is NO effect, relation, or difference between what is being investigated. This is known as the null hypothesis. Next, the researcher evaluates the probability of data, given this null hypothesis. Consider a research team investigating the relation between drinking coffee and hating one’s boss. The team begins with the null hypothesis, that coffee has NO effect on how much someone hates their boss. Then the team determines the probability of data (or more extreme data), assuming the null hypothesis is true.

More technically, significance testing uses an index called the *p*-value to determine if a result is statistically significant. Specifically, beginning with the assumption that the true effect is zero (i.e., the null hypothesis is true), a *p*-value indicates the proportion of test statistics, computed from hypothetical random samples, that are as extreme, or more extreme, than the test statistic observed in the current study. If a *p*-value is low, it indicates that, when the null hypothesis is true, a small number of results would be as extreme or more extreme than the current result. Traditionally, statistical significance is declared if less than 5% of other results would be more extreme than the result observed in the current study, when the null is true (i.e., when there is no effect). This 5% cut-off corresponds to setting the alpha-level (Type I error rate) to 0.05. Though most researchers use an alpha = 0.05 level by convention, some have put forth that a much lower convention for alpha (i.e., alpha = 0.005) is needed to obtain results that replicate; whereas others have suggested that researchers should have the flexibility to set alpha on a case by case basis (c.f., Benjamin et al., 2018; Lakens et al., 2018). Historic convention, and historic convention only, explains the default 0.05 level. To summarize, statistical significance indicates that a small number of other hypothetical results (typically less than 5% from a very large number of hypothetical results) would be as extreme or more extreme than what was observed in the current study, when it is assumed that the null hypothesis is true.

With its many technicalities, significance testing is not inherently ready for public consumption. It involves conditional probability, hypothetical results (whatever those are), and the null hypothesis (a peculiar starting assumption given researchers are often examining relations for the very reason that they expect them to be non-zero). Is there a way to bypass the technical details and hypotheticals, but still accurately convey what statistical significance means? We think that there is. To do so, we consider the end utility of significance testing and leverage this deduction rather than trying to parse the technical aspects of its definition into something palatable and easily digestible.

## May Not Be Zero

According to the correct definition of statistical significance, what is the end utility of concluding that a result is statistically significant? We propose that the utility may be seen as follows: Given that there seems to be a low probability of getting results as extreme, or more extreme, than what was observed when I assume the actual effect is zero (i.e., the data are unlikely, given the null) perhaps my starting assumption that there is no relation is incorrect. In other words, concluding that something is “statistically significant” is not dissimilar from saying, there is now some reason to believe that the effect is non-zero. I cannot say what it is, it just may not be zero. Effect sizes and confidence intervals can give information about what the effect may be, but statistical significance alone does not provide information about how large an effect may be – it just MAY not be zero.

We suggest that this, “may not be zero,” interpretation is a simple, concise, and not incorrect interpretation of statistical significance. We can put this interpretation into practice by applying it to the opening paragraph’s trilemma:

***Hypothetical popular press editor’s suggestion:***

“All of the results were statistically significant (indicating that results were not likely due to chance).”

***Changed to:***

“All of the results were statistically significant (indicating that the true effects may not be zero).”

Or

“All of the results were statistically significant (which suggests that there is reason to doubt that the true effects are zero).”

## Challenges and Limitations

What is clear form this interpretation is that it is uninformative, bordering on meaningless. This is true and this is the nature of significance testing. Attempts to get more interpretational juice from the proverbial squeeze when interpreting statistical significance are likely lead to interpretational overreach and predictable mistakes. If information beyond “may not be zero” is desired, researchers should supplement *p*-values with other types of statistical information to avoid making incorrect inferences from statistical significance. The American Statistical Association statement on *p*-values indicates, “a *p*-value, or statistical significance, does not measure the size of an effect or the importance of a result” (p. 132; Wasserstein and Lazar, 2016). The American Statistical Association also notes that “Scientific conclusions and business or policy decisions should not be based only on whether a *p*-value passes a specific threshold” (p. 132). Accordingly, effect sizes with confidence intervals can be used to give readers at least an estimate of the magnitude of the effect being investigated. That said, confidence intervals are not without interpretational challenges (Cumming et al., 2004; Fidler et al., 2004; Hoekstra et al., 2014, Morey et al., 2016). If researchers desire additional information from their analyses, techniques that include Bayes factors and credibility intervals should also be considered as desirable alternatives (to learn more see Gelman et al., 2013; Kruschke, 2014; McElreath, 2016; Etz et al., 2018).

What if a result was not statistically significant? Does that at least tell us that the null hypothesis is true? Sadly, no. Because significance testing assumes the null is true, *p*-values only provide information against the null hypothesis and not in favor of it. Therefore, even though it was assumed the null is true, failing to find statistical significance also fails to provide information about the accuracy of this assumption. If researchers are interested in examining the probability that the null hypotheses is true (or at least more likely than an a specified alternative hypothesis) Bayesian techniques can be informative (e.g., Gelman et al., 2013; Kruschke, 2014; McElreath, 2016). Consequently, failing to find statistical significance leaves one in a position not dissimilar from finding statistical significance. In both cases, the true effect still may not be zero.

## Conclusion

Researchers in applied fields like I/O psychology are often required to communicate and interpret what statistical significance means to non-scientific audiences. Relying on a technically accurate formal definition of statistical significance is not always productive because it is not meaningful or intuitive for general audiences. Properly understanding technically correct definitions is challenging even for trained researchers, as it is well documented that statistical significance is frequently misunderstood and misinterpreted by researchers who rely on it (Nickerson, 2000; Wasserstein and Lazar, 2016). Close to a century’s worth of research and its application shows that when researchers interpret statistical significance they make mistakes. Having a short, simple, and correct interpretation of statistical significance, like the one we provided, may help researchers avoid making mistakes when they need to communicate what statistical significance means.

Significance testing can be a helpful tool for making inferences from data. However, as is the case with other useful tools, mistakes and accidents sometimes happen when using the tool. This is why so many useful tools have safety features added to them over time to prevent accidents from mistakes or probable habits of misuse (e.g., firearms have safeties, chainsaws have chain brakes, etc.). Statistical significance has been used for a long time without the aid of safety features to deter inappropriate use and avoid accidents. The short hand interpretation we provide (i.e., interpreting statistical significance as “may not be zero,”) can be viewed as a safety feature that may reduce science communication accidents when significance testing is used when communicating with the general public. Our short-hand interpretation also has a clear advantage of making it readily apparent how uninformative significance testing is on its own. This makes it hard to oversell and overstate the importance of single research findings and allows practitioners and consumers of research to have an honest accounting of what research is telling them.

## Author Contributions

JS and DS wrote the paper.

## Conflict of Interest Statement

The authors declare that the research was conducted in the absence of any commercial or financial relationships that could be construed as a potential conflict of interest.

## Footnotes

1 http://www.apa.org/science/leadership/bsa/statistical/
